# Surface Free Energies and Entropy of Aqueous CaCO_3_ Interfaces

**DOI:** 10.1021/acs.langmuir.4c04738

**Published:** 2025-03-18

**Authors:** Emma Armstrong, Stephen R. Yeandel, John H. Harding, Colin L. Freeman

**Affiliations:** † Department of Materials Science and Engineering, Sir Robert Hadfield Building, University of Sheffield, Mappin Street, Sheffield S1 3JD, U.K.; ‡ Information School, The Wave, University of Sheffield, 2 Whitham Road, Sheffield S10 2AH, U.K.

## Abstract

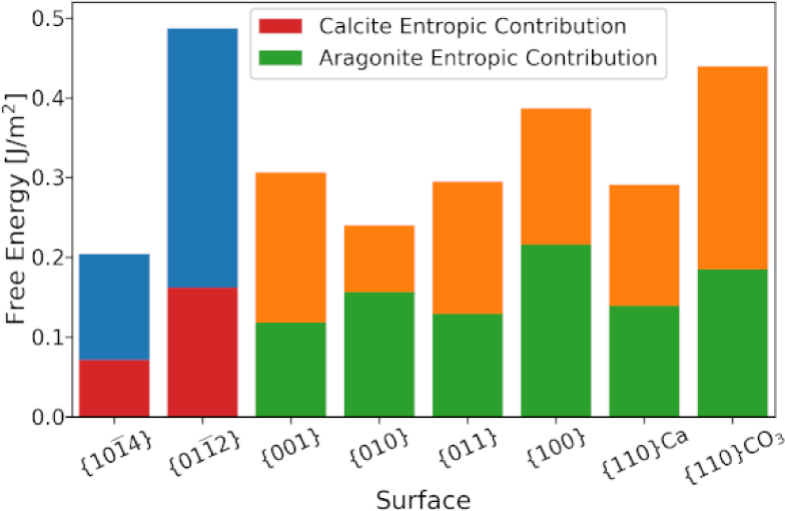

This work uses a recently proposed methodology to calculate the free energies of calcite and aragonite interfaces with water. This method properly includes the entropic contributions, ignored or approximated in previous work. By including this entropic component, we show that the aqueous calcite {101̅4} surface has a lower free energy than any of the aragonite surfaces. This resolves the discrepancies in previous simulation work that suggested that an aragonite nucleus would be more stable than a calcite one. Our analysis of the water structure highlights the generally greater entropic contribution to the interfacial free energy at the aragonite/water interface than at the calcite one. These methods are applied to a range of temperatures to examine how the solution temperature alters the interfacial energies. Our results are then discussed in the context of calcium carbonate nucleation and polymorph–morphology selection under different environmental conditions.

## 1 Introduction

The nucleation and growth of calcium carbonate-based minerals remains a key area of interest to many communities. Within nature, CaCO_3_ is produced as a biomineral by many organisms, making it a key factor in the ocean’s carbon budget.^1^ This natural production of CaCO_3_ demonstrates a control over morphology and phase that is difficult to replicate in laboratories.^2^ Industrially, CaCO_3_ is used extensively as a whitening agent, but also, due to its natural abundance, it is a problem for scaling in pipes where its formation needs to be limited and controlled.^3^ This has led to a continued interest in understanding the growth, dissolution, and phase selection of this material.^4−6^ At the center of nucleation and growth is the mineral/water interface. In classical nucleation theory, the energy of the interface created between the nucleus and its surrounding environment is the key component of the barrier to crystal formation since the nucleation rate depends on the exponential of its cube. Under pure thermodynamic control, the size of this interfacial energy will control the facets of the growing crystal and potentially influence the phase selection through its contribution to the total free energy of the nuclei. The interfacial energy can also direct much of the chemistry and physics of the material, such as adsorption and corrosion. This has led to extensive research into methods of estimating its value, modeling the behavior at the interface and using it to predict phase formation.^7−10^

For CaCO_3_, the calcite/water interface has been widely studied,^10−14^ and both experimental and simulation methods have shown clear evidence of a strong interaction between the mineral ions and the surface water. There have been many attempts to use atomistic simulations to calculate the interfacial energies of formation of calcium carbonate polymorphs with water. de Leeuw and Parker^15^ used static simulations to calculate the *configurational* energies of formation for various calcite and aragonite hydrated interfaces. These were assumed to be a good approximation of the internal energies and (ignoring the entropies of formation) the Helmholtz free energies. The calcite {101̅4} interface was undoubtedly the most stable of all of the interfaces they considered. The equilibrium hydrated morphology of calcite is therefore dominated by a single interface,^16−18^ and its calculated morphology, determined with the Wulff construction,^19^ strongly resembled the experimental morphology. The same was not true for aragonite. Here, a range of accessible energies was found, and more interfaces were expressed. The calculated morphology still showed some resemblance to the observed mineral morphology, but it was a poorer match to the experiment than for the calcite case. More recent work by Bano et al.^12^ implemented newer force-fields^20^ and simulation methods to reconsider the values for the calcite and aragonite interfacial energies. The values obtained, however, were nearly all negative and small in magnitude. This suggests that the bulk crystal should be unstable in aqueous environments. Sun et al.^21^ reported aragonite surface energies obtained using density functional theory (DFT) that gave similar values for different surface cuts, but their numbers also suggested that ∼20% of the calcite surface should be the {101̅0} surface (rather than purely {101̅4}), which disagrees with all experimental work. This result is probably due to the small system sizes imposed by DFT that limit the number of surface waters available to stabilize the interface. The use of implicit water models in classical simulations^22^ gave rise to similar problems, demonstrating the importance of explicitly modeling enough water molecules.

It is well-known from both simulation and experiment that the water structure close to an interface with a mineral is often profoundly altered from the bulk. The mineral surface imposes structuring on the water, which extends out from the interface. This structuring will lead to a loss of entropy of the water molecules compared to that of the bulk system. Freeman and Harding^14^ have previously estimated the amount of entropy loss for each water molecule adsorbing to the calcite {101̅4} surface using thermodynamic integration (TI). They obtained a value of ∼6 J mol^–1^ K^–1^. In their discussion, Bano et al.^12^ argued that the structured water layers found at the crystal–water interface could be considered as ice-like and proposed that the entropy of fusion in the ice-liquid water system could be used to approximate the entropy contribution to the interfacial free energy of formation. This gave a positive contribution that was large enough to compensate for the negative configurational interfacial energies. The results of their study, however, suggest that some of the aragonite surface energies are lower than the {101̅4} calcite surface, and so, following nucleation theory, we would expect to observe an aragonite nucleus before a calcite one. Experimentally, calcite dominates the mineral growth, and substantial quantities of aragonite are only observed at solution temperatures in excess of 70 °C.^23^ The formation of aragonite in higher temperature solutions has been linked to the structure of amorphous calcium carbonate (ACC) intermediates^6,24^ but remains unexplained. Alternatively, aragonite is formed via the use of additives such as Mg ions^5,6^ or functional molecules.^25^ The effect of the additives has been explained as the altering of the surface energies by attachment, which may inhibit calcite growth or encourage aragonite growth.^25^ This highlights the importance of the interfacial energy. The discrepancy in CaCO_3_ interfacial energies^12^ may come from the assumption that the entropy of formation is the same for all interfaces although each surface may generate different ordering. Previous simulations of calcium sulfate surfaces have shown a significant difference between entropy changes for water at different surfaces of gypsum and bassanite.^9^

It is clear that a full calculation of the interfacial energy of the calcium carbonate system is required. In this work, we will employ a method that uses Einstein crystals as a thermodynamic reference^9^ to calculate the interfacial free energies of aqueous calcite and aragonite surfaces. This includes the entropy terms and we will show that they are both too large to be ignored and differ too much from interface to interface to be estimated by a single universal correction.

## 2 Methodology

The interfacial free energy, γ_Solid+Liquid_, between a solid and liquid phase is defined as the reversible work required to form a unit area of the solid/liquid interface. The interfacial free energy is challenging to calculate from simulations when dealing with mixed-phase systems. Frequently, the configurational interfacial energy (usually taken to be a good approximation to the interfacial enthalpy) is computed instead and used in determining the crystal stability and morphology. A range of different computational techniques is available. The “cleaving wall”^26,27^ process requires the introduction of a potential function that separates the liquid and solid components followed by a deactivation of interactions. These separated units are then reformed into their bulk components. This creates a reversible pathway necessary for a free energy process. The “mold integration” method uses a mold of potential wells to generate a solid slab within the fluid phase.^7^ Further alternatives include contact angle simulations^28^ and the use of metadynamics.^29^ A recent review of methods to calculate free energies of solid/liquid interfaces can be found in Di Pasquale and Davidchack.^30^ We have made use of the Einstein crystal method due to its general applicability to a variety of surface problems. It is not limited to nonpolar or flat well-defined surfaces, nor does it require particularly large simulations or the identification of order parameters to direct the conversion or the study of thermodynamic states where we are near a phase transition. The methods are briefly discussed below.

### 2.1 Calculating Interfacial Free Energies Using Einstein Crystals

Interfacial free energies are not absolute free energies but are defined with respect to the bulk systems with no interface present (if we consider the interfacial free energy with respect to the free surfaces of water and calcium carbonate, we refer to it as a cleavage free energy). We can therefore choose a common reference state to which both the bulk and interfacial systems can be easily transformed. In our recently developed Einstein crystal method,^9^ the common reference state was chosen to be the Einstein crystal. This has the useful property that the absolute Cartesian coordinates of the atoms do not affect the free energy, which significantly simplifies the calculation of interfacial free energies because explicit real-space rearrangement of atoms can be entirely avoided. Thus, the method can be applied to much more complex systems than previous methods. Throughout this paper, we use the symbol  to denote the free energy of a process α → β. A similar notation is used for other thermodynamic quantities.

The Einstein crystal method makes extensive reuse of previously calculated values to optimize efficiency. The framework can accommodate both dipolar surfaces and miscible molecules at the interface. As we are studying the anhydrous phases of calcium carbonate, however, the miscible species correction discussed in Yeandel et al. is not required. We start with a single thick slab of liquid with a vacuum gap in the simulation cell. Two liquid/vacuum interfaces are also present in the system. If a large enough water slab is used, then the solid–liquid interface will not change this interface between different systems, and so their contribution to the interfacial free energy will be zero. Convergence testing of the water slab can confirm this. We then split the liquid slab in two, generating a second vacuum gap with a free energy cost of . We insert a solid slab into this gap, producing two solid/liquid surfaces, costing a free energy of . The interfacial free energy of the solid–liquid interface, γ_Solid+Liquid_, is then:
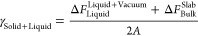
1where *A* is the area of the interface. We can obtain the free energy of transforming the bulk solid into a slab by transforming both into an Einstein crystal and using the identity:
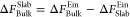
2which enables us to write:
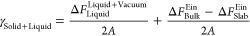
3

The first term can be identified with the surface free energy (surface tension) of the liquid, γ_Liquid_, which can be calculated separately using, for example, the method of Kirkwood and Buff.^31^ The second term may be calculated by TI as discussed in detail in Yeandel et al.^9^

The calculation of the enthalpy of formation of the interface, Δ*H*_Solid+Liquid_, is much simpler. Formally, we can write this as follows:
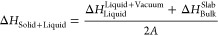
4A transformation to an Einstein crystal is unnecessary. The enthalpy of the components of the system—the bulk, slab, liquid, and liquid/vacuum interface—can be calculated with respect to the standard reference state of the individual species at rest at infinity.
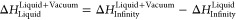
5
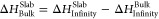
6

With both the interfacial free energy and interfacial enthalpy known, the entropy, Δ*S*, of the system can be simply calculated using Δ*S* = (Δ*H* – Δ*F*)/*T*. Full details can be found in the original paper by Yeandel et al.^9^

### 2.2 Simulation Setup

For calcite, the {101̅4} and {011̅2} interfaces were considered. For aragonite, {001}, {010}, {011}, {100}, {100}Ca, and {100}CO_3_ were investigated. Each interface calculation followed the procedure outlined in Section 2.1. All simulations were performed using the Large-scale Atomic/Molecular Massively Parallel Simulator (LAMMPS) program.^32^ The force-fields of Raiteri et al.^33^ were used for CaCO_3_ and the SPC/Fw force-field for water.^34^

**Figure 1:**
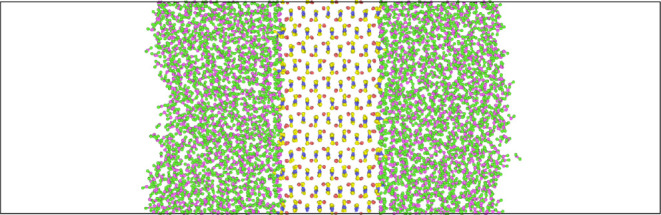
Initial simulation setup for an example system. The water has been introduced to the system during a simulation run. The vacuum gap on either side is large enough to ensure that no significant interactions occurred across the boundary.

The initial configuration is depicted in Figure 1. The number of water molecules used was selected to create a roughly equal water layer thickness (∼30 Å) across all surfaces. Both the solid slab and the water layers are wide enough to ensure that interactions are not occurring across the slab, and the “bulk” water behavior is present as well as the surface effects.

The simulation cell is then equilibrated by an NPT run where the simulation cell vector perpendicular to the slab orientation is held constant. Initially, simulations were performed at 300 K and 0 bar using the Nosé–Hoover thermostat and barostat with relaxation times of 0.1 and 1.0 ps, respectively, and a 1 fs time step. The long-range electrostatic interactions were determined using a PPPM algorithm^35^ with a relative force accuracy of 1.0 × 10^–5^. The cell is equilibrated for 100 ps, and the cell lattice vectors are calculated over the remaining 500 ps run. A short NVT run is then performed to ensure the stability of the cell before TI is performed.

Two TI calculations are required to convert the solid to an Einstein crystal: one to activate the harmonic wells in the solid and the other to turn off all interactions for the solid atoms. This splitting of the pathway results in a more stable transformation to an Einstein crystal than changing both types of interaction simultaneously. For the first calculation, the harmonic wells are gradually switched on as λ varies between 0 and 1, while the other atom interactions are still present. For the second calculation, the harmonic wells remain fully switched on, and the other potentials are switched off as λ varies from 1 to 0. Each calculation consists of 100 discrete values of λ to ensure a smooth integration curve. The MD simulations for each λ value were performed in parallel for 0.5 ns each using a Langevin thermostat. The Langevin thermostat is employed here as the Nosé–Hoover thermostat does not provide full ergodic sampling at the end of the integration pathway when the system is almost purely composed of harmonic oscillators.^36^ The potential energy is sampled every 1 ps.

A similar procedure is followed for the bulk phases of calcite and aragonite but without the inclusion of water or the vacuum. All of the cell vectors are unconstrained in the equilibration run. The separate pathways are numerically integrated, and the difference between the energies is the free energy associated with the conversion of the slab or bulk into an Einstein crystal, , required for computing the interfacial free energy. The results obtained for  for calcite and aragonite can be rescaled and reused for each interfacial free energy calculation of the respective interfaces with eq 3. Additionally, the value of γ_Water_ may be reused for all calculations at 300 K. The surface tension of water has been taken from the original paper^9^ as 0.0581 J/m^2^ since the same water model is being used.

The same procedure was followed at various temperatures to calculate the interfacial free energy of the mineral surfaces over a temperature range of 280–450 K. The melting and boiling points of the water model SPC/Fw^34^ do not align with the known experimental temperatures for water. In fact, the melting and boiling points for our chosen model are not well characterized. For the rigid water model SPC/E, the melting and boiling temperatures are quoted as 215^37^ and 396 K,^38^ respectively. Although we are not interested in the behavior at the transition points, we are interested in the temperature region in which aragonite becomes favored over calcite (experimentally ∼330–350 K). Given that the transition points are unknown for the water model, this temperature range for aragonite formation is also unknown. The boiling point quoted is not much larger than the known value for water; however, other estimates and other models predict substantially higher values. Therefore, we have elected to expand our temperature range in the hope of encapsulating the surface behavior around the point at which aragonite is favored.

Calculated surface free energies (surface tensions) of water from 280 to 450 K are shown in Figure S1. The values show an approximately linear dependence on temperature, in line with expectations.^39^ Experimentally, the surface tension of water at 300 K is 0.072 J/m^2^ (72 mN/m), slightly larger than our value of 0.058 J/m^2^. It is known that our chosen water model underestimates the surface tension,^40^ but, since variations of the computed and experimental results with temperature are similar, our results are good enough for use in further calculations. The bulk water enthalpy increases as the temperature rises (Figure S1). On the other hand, the surface enthalpies do not vary linearly as the temperature increases. The surface enthalpy increases until around 360 K and becomes constant between 400 and 450 K. The calculated values can be reused in all calculations performed at the same temperature. In our simulations, the water–vacuum interface remained stable over all the temperatures studied.

### 2.3 Analyzing the Solvent Ordering

Ordering of water at the calcite interface has been reported both experimentally and computationally,^13,20,41^ and structured layers of water molecules are observed at the interface. For aragonite, less is known about the mineral/water interface. In the previous work of Bano et al.,^12^ a similar degree of ordering to calcite was identified. The task of quantifying order is not trivial, and there is no perfect method. In principle, there are many factors to consider: translational movement, rotational movement, molecular orientation, and time or distance correlations. The Steinhardt order parameters are commonly used as these reveal local coordination order around an atom or molecule, which can be used to distinguish between structure types.^42^ A valuable discussion of their use in water structuring at the surface of TiO_2_ can be found in the paper by O’Carroll and English.^43^ This process is useful for examining localized water–water structure but does not necessarily provide much information about reductions in configurational space that the water molecules can explore.^14^ Therefore, it may not be a useful guide to the entropy loss of the water molecules at the surface. We therefore attempted to explore ordering through density analysis.

#### 2.3.1 Density Profiles and the Spatial Ordering Parameter

One of the simplest ways of observing order is the density profile in the direction normal to the plane of the slab (usually called the *z*-density profile, ρ(*z*)). The position of a water molecule is taken to be that of the oxygen atom, *O*_W_, and the *z*-density profile is constructed with the outermost calcium ion of the nearest interface taken as the origin of the *z*-axis.

The *z*-density profile gives no information about ordering in planes parallel to the interfacial plane. For these purposes, the density across the *zx* and *zy* planes is calculated. Regions of both high and low densities of water molecules are present in most of the systems. As the bulk water has a uniform density, any deviation from this distribution can be ascribed to water ordering. A 6 Å thick slab of water at the interface is compared to a slab of equal width 12 Å from the interface, where the water shows bulk behavior. We then calculate an order parameter, *f*_order_, defined as follows:
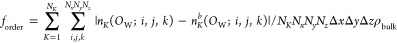
7where *n*_*K*_(*O*_W_; *i*,*j*,*k*) is the number of water oxygen atoms in the *i*,*j*,*k* cell for the interface and (*O*_W_; *i*,*j*,*k*) is the number of water oxygen atoms in the equivalent cell of bulk water in the *K*^th^ configuration. This cell has volume Δ*x*Δ*y*Δ*z* where Δ*x* = *L*_*x*_/*N*_*x*_, Δ*y* = *L*_*y*_/*N*_*y*_, and Δ*z* = 6.0/*N*_*z*_ Å. *N*_*K*_ is the number of configurations considered in constructing the order parameter. A small value for *f*_order_ therefore suggests that the interfacial water structure is similar to that of the bulk. A greater value of *f*_order_ suggests greater deviation from the bulk, and therefore, that more ordering has occurred.

With the above method, there is a significant dependence of the final value on the chosen bin size. To select an appropriate bin size, convergence tests were run on a separate system of bulk water. The number of frames sampled that are required for the bulk system to be considered to be homogeneous was calculated. A measure of the homogeneity is obtained by calculating the standard deviation of the water density across planes of the system and then normalizing by bin area; a low standard deviation suggests a more constant density across the system and thus a more homogeneous system. A bin size of 0.25 Å was used for the interfacial systems as an acceptable compromise between computational cost and homogeneity.

#### 2.3.2 Hydrogen Bonding

Another way of determining the behavior of the water at the interface is to look at the hydrogen bonding in the water. We can see whether the amount of hydrogen bonding at the interface is significantly different from the bulk hydrogen bonding to identify possible ordering. For this work, we use the criterion first proposed by Haughney et al.^44^ where the distance between the donor and acceptor oxygen atoms *r*_OO_ < 3.5 Å and the angle between the donor oxygen, hydrogen, and acceptor oxygen θ < 30°. The average number of hydrogen bonds per water molecule in bulk water is calculated and used for comparison. At 300 K, the average number of hydrogen bonds is computed as 1.795 per molecule. A slab of water from the interface 6 Å wide defines the interfacial waters, as this captures most of the ordering.

## 3 Results and Discussion

### 3.1 Interfacial Free Energies of CaCO_3_ and Water

Results for the interfacial free energies, comprising both enthalpy and entropy of the selected calcite and aragonite interfaces, are given in Table 1. As with previous studies and experimental findings, the calcite {101̅4} interface has unequivocally the lowest interfacial free energy (0.21 J/m^2^) and is thus the most stable interface. The interfacial surface free energies presented here are calculated with reference to bulk crystals and bulk water.

**Table 1:** Interfacial Free Energies at 300 K and The Individual Enthalpy and Entropy Components^a^

Interface	Free Energy Δ*F* (J/m^2^)	Enthalpy Δ*H* (J/m^2^)	Entropy Δ*S* (μJ/m^2^/K)
Calcite			
{101̅4}	0.205	0.133	–237
{011̅2}	0.487	0.325	–540
Aragonite			
{001}	0.307	0.189	–393
{010}	0.240	0.084	–520
{011}	0.295	0.166	–430
{100}	0.387	0.171	–720
{110}Ca	0.291	0.152	–463
{110}CO_3_	0.440	0.255	–617

a Δ*F* and Δ*H* are obtained from MD simulations and Δ*S* from the difference between them.

The {011̅2} interface has the highest free energy of any slab considered. For the {011̅2} interface, the dipole across the slab was removed in the cutting process by translating calcium ions from the top surface to the bottom. A second configuration with the dipole has been calculated; however, the resultant energy was much larger, 1.10 J/m^2^, and it has not been considered in further analysis. Bruno et al.^22^ reported a value of 0.49 J/m^2^ for the free energy of the {101̅4} interface with water using static simulations. Notably, this value is dependent on experimental values of the ion hydration energy and the ionic radii, which have been further updated.^45^ More recently, the values of 0.16 J/m^2^ and 0.18 J/m^2^ have been calculated by Bano et al.,^12^ in good agreement with our results. Since the work reported above and our own work used different calcium carbonate force-fields, there are inevitably small discrepancies in the values.

There have been many attempts to obtain an experimental value for the solid–liquid interfacial free energy. A selection of them is shown in Table 2 together with a brief indication of the method used to obtain them. Two measurements of the heat of immersion (*q*_imm_ = γ_Solid+Liquid_ – γ_Solid+Vapor_) have been omitted^46,47^ since they imply a negative value for γ_Solid+Liquid_ for any reasonable estimate of the surface/vapor free energy, γ_Solid+Vapor_. Also, the value of Costa et al.^48^ should be omitted since it relies on the dubious estimate of the free energy given by Bruno et al.^22^ Wang et al.^49^ have also argued that the value of Forbes et al.^50^ is far too large to be compatible with the rates of nucleation observed. Indeed, it is so large that the nucleation of calcite would never be seen.

**Table 2:** Solution–Solid Interfacial Free Energies (Except for Ref (50) which is an enthalpy) in J/m^2^ for CaCO_3_ (calcite)^a^

	γ_Solid+Liquid_ (J/m^2^)	Face	Technique
Söhnel and Mullins 1982^51^	0.098	average	expt: homogeneous nucleation
Liu and Lim 2003^52^	0.170	average	expt: homogeneous nucleation
Røyne et al. 2011^53^	0.150	(101̅4)	expt: subcritical cracking
Jańczuk et al. 1986^54^	0.098	(101̅4)	expt: contact angle
Okayama et al. 1997^55^	0.072	(101̅4)	expt: contact angle
Costa et al. 2018^48^	0.410	(101̅4)	expt: contact angle
Hadjittofis et al. 2021^56^	0.055	(101̅4)	expt: inverse gas chromatography
Forbes et al. 2011^50^	1.480 ± 0.21	average	expt: calorimetry
This work	0.205	(101̅4)	calc: free energy

a Only values for the (101̅4) interface are quoted. If the term “average” is used, it reports a measurement on a powder (or the analysis of homogeneous nucleation data). In such cases, the average is still dominated by the value for the (101̅4) interface. If a temperature is quoted, it is 298 K; otherwise, it is said to be at “room temperature″.

Even if we discard some values as suggested above, the spread of possible values is still wide (0.055–0.170 J/m^2^). Our calculated value for the free energy at 298 K is higher than any of them except Costa.^48^ The reference state for the free energy is likely to be a key factor in the energetic differences. Our simulations are calculated with reference to a bulk crystal and bulk water, while values extracted from nucleation studies will be referenced to ions in solution. The values we have calculated also refer to a flat infinite surface whereas it is likely that both the contact angle experiments and the results from homogeneous nucleation (analyzed using classical nucleation theory) require corrections that take account of the curvature of the interface (see Hijes^57^ for a detailed discussion) and may be affected by the presence of screw dislocations. Therefore, we are likely to produce a larger value where the extra relaxations seen in the experiment are not present.

For aragonite, there is much less difference between the interfacial free energies. Although the {010} interface is the most stable, it is less stable than the calcite {101̅4} interface. The {001}, {011}, and {110}Ca interfaces all lie within 0.016 J/m^2^ of each other, and any slight variation in the free energy values could alter their relative stability. The carbonate-terminated {110}CO_3_ interface has a much higher interfacial energy than its calcium-terminated counterpart, {110}Ca, and hence, the latter is likely to be favored in the resulting crystal morphology. The {100} interface of aragonite has the next highest free energy. Despite the higher number of interfaces considered, the range of values for all of the aragonite interfaces (0.440–0.240 J/m^2^) is less than that between the two calcite surfaces (0.487–0.205 J/m^2^). The bunching of the aragonite interfacial free energies is reflected in the variety of aragonite morphologies and expressed surfaces observed when precipitated experimentally. Although values for the interfacial energies of the numerous aragonite/water interfaces are available,^15,21,58,59^ there is very little on the interfacial *free* energies. The most stable calculated interfaces, {010} and {110}, however, are the faces more often expressed in naturally formed aragonite.^16,17^ Values for the interfacial free energies for two (unidentified) aragonite solid/solution interfaces have been measured by Hadjittofis.^56^ They quote values of 0.042 and 0.054 J/m^2^. These are similar to their calcite values and much smaller than our calculated values. It is likely that once more, this is due to effects of interface curvature.

### 3.2 Crystal Morphology

**Figure 2:**
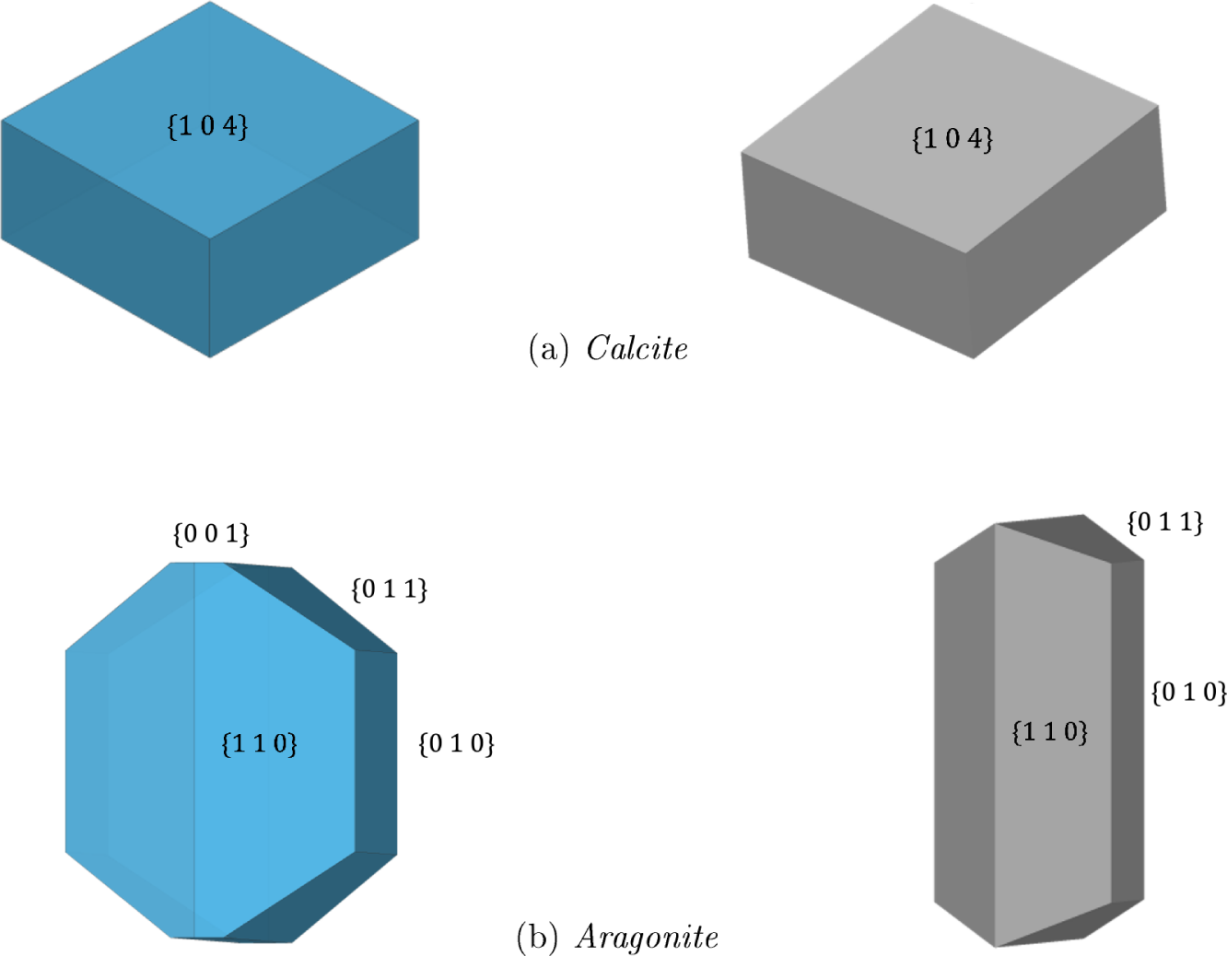
Crystal morphologies of (upper left) calcite (a) and (lower left) aragonite (b) in pure water at 300 K calculated with the Wulff construction method and compared with images constructed from their respective measured equilibrium morphologies (right) using data from previous studies.^16,17^

Using the interfacial free energies in Table 1, the Wulff construction^19^ can be used to determine the equilibrium morphology of calcite and aragonite nanoparticles in pure water. The shape of the nanoparticle is determined by minimizing the overall free energy. Our hydrated morphologies for calcite and aragonite are shown in Figure 2 alongside the known equilibrium structures.^16,17^

The rhombohedral morphology of calcite (Figure 2a, left) agrees with previous morphological predictions and experimental morphologies. Only the {101̅4} surfaces are expressed.^15,18^ The aragonite morphology (Figure 2b, left) shows a strong resemblance to the equilibrium morphology, particularly in comparison to previous morphology predictions.^15,58^ Despite not appearing in the equilibrium morphology, the {001} interface has been observed experimentally^60^ and is therefore not inconceivable as part of the structure. Generally, scanning electron microscopy images show hexagonal rods or needles of aragonite,^18,61,62^ but there are many factors that could alter our predicted morphologies in the direction of those seen; kinetic effects are ignored here as well as the presence of impurities.

### 3.3 Relative Nanoparticle Stabilities

Given the interfacial free energies, we use the Wulff construction to describe the resultant nanoparticle stability. We follow the same process as Yeandel et al.^9^ The experimental free energy of conversion of aragonite to calcite, , is −840 ± 20 J/mol at 298 K^63^ (used in the fitting process for a calcium carbonate force-field by Raiteri et al.^20^) Our value for the free energy difference is −612 J/mol, but the force-field used was the slightly later one.^33^ Values of 1.28 and 1.14 were obtained for the shape factors of calcite and aragonite, respectively. As only the {101̅4} face is expressed in the calcite morphology, the weighted average interfacial free energy is simply the interfacial free energy of the surface, γ_Nano_ = 0.20 J/m^2^, whereas for aragonite, the weighted average value is γ_Nano_ = 0.28 J/m^2^. The relative free energy of an aragonite nanoparticle with respect to a calcite nanoparticle is always positive, meaning calcite is always the favored polymorph regardless of the number of formula units, in line with experimental findings. A negative value would suggest that an aragonite nanoparticle is more likely to form.

### 3.4 Enthalpy and Entropy Values

In addition to the interfacial free energy, the interfacial *enthalpy* of each slab has been calculated with eq 4. The enthalpies of the slab, , and the corresponding bulk, , were obtained from molecular dynamics runs under the same conditions as the TIs but with the harmonic wells turned off and the interatomic potentials on. Values for the bulk water enthalpy,  (−42.2 kJ/mol per formula unit), and the water/vacuum surface enthalpy,  (0.115 J/m^2^), were taken from Yeandel et al.^9^

The interfacial entropy, Δ*S*, is obtained from the standard identity.
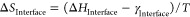
8

**Figure 3:**
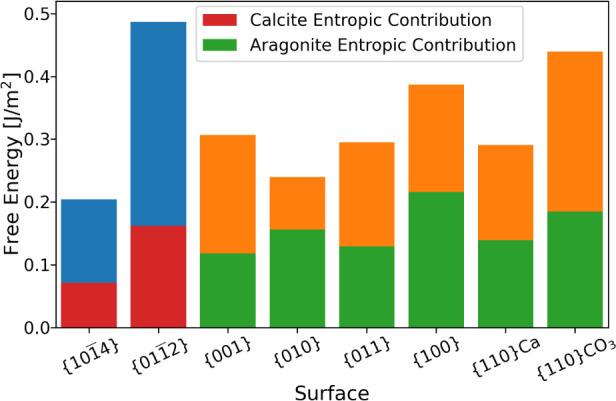
Interfacial free energies Δ*F* of all surfaces at 300 K. The entropy contribution is associated with the formation of the interface (i.e., solvent and solid). −*T*Δ*S* is also displayed for each surface, and the remaining portion of the free energy is the enthalpic contribution Δ*H*.

The values are shown in Table 1. The *entropic* contribution to the interfacial free energy, , is displayed in Figure 3 for 300 K.

One of the first things to note is that the entropic contribution is not uniform across all of the interfaces, showing that a single correction for all interfaces is not good enough when considering stability, particularly in the case of calcium carbonate where there is very little separating the thermodynamic stability of the two phases. Second, the ratio of entropic contributions to the total free energies of the aragonite interfaces is much greater than those for calcite. For the {101̅4} and {011̅2} interfaces of calcite, 35% and 33% of the free energy is due to the entropy, which is lower than for any of the aragonite surfaces. The contributions for the aragonite interfaces vary from 39% to 65%, with the {010} and the {110}Ca surfaces having only small enthalpic contributions. For example, the enthalpy of the {010} aragonite/water interface is lower than that of the {101̅4} calcite/water interface. Therefore, simulations neglecting the entropic contribution (or assuming they were the same for all surfaces^12^) could result in the conclusion that aragonite gave the most stable nuclei for calcium carbonate. Our methods remove this disagreement with experiment as the large entropic contribution results in a less stable aragonite interface. If the magnitude of the entropy was reduced, say by impurities disrupting the water structuring, the aragonite surface would become more stable, presenting a possible means of promoting aragonite over calcite nucleation.

The small change in enthalpy is due to the strong binding of water with the Ca^2+^ ions at the surface. This accumulation of molecules at the surface imposes water ordering at the interface, which results in an entropy penalty compared to the disorder of bulk water. Therefore, for more information on the role entropy contributes to the interfacial free energies, analysis of the water at the surface is required.

### 3.5 Water Ordering

**Figure 4:**
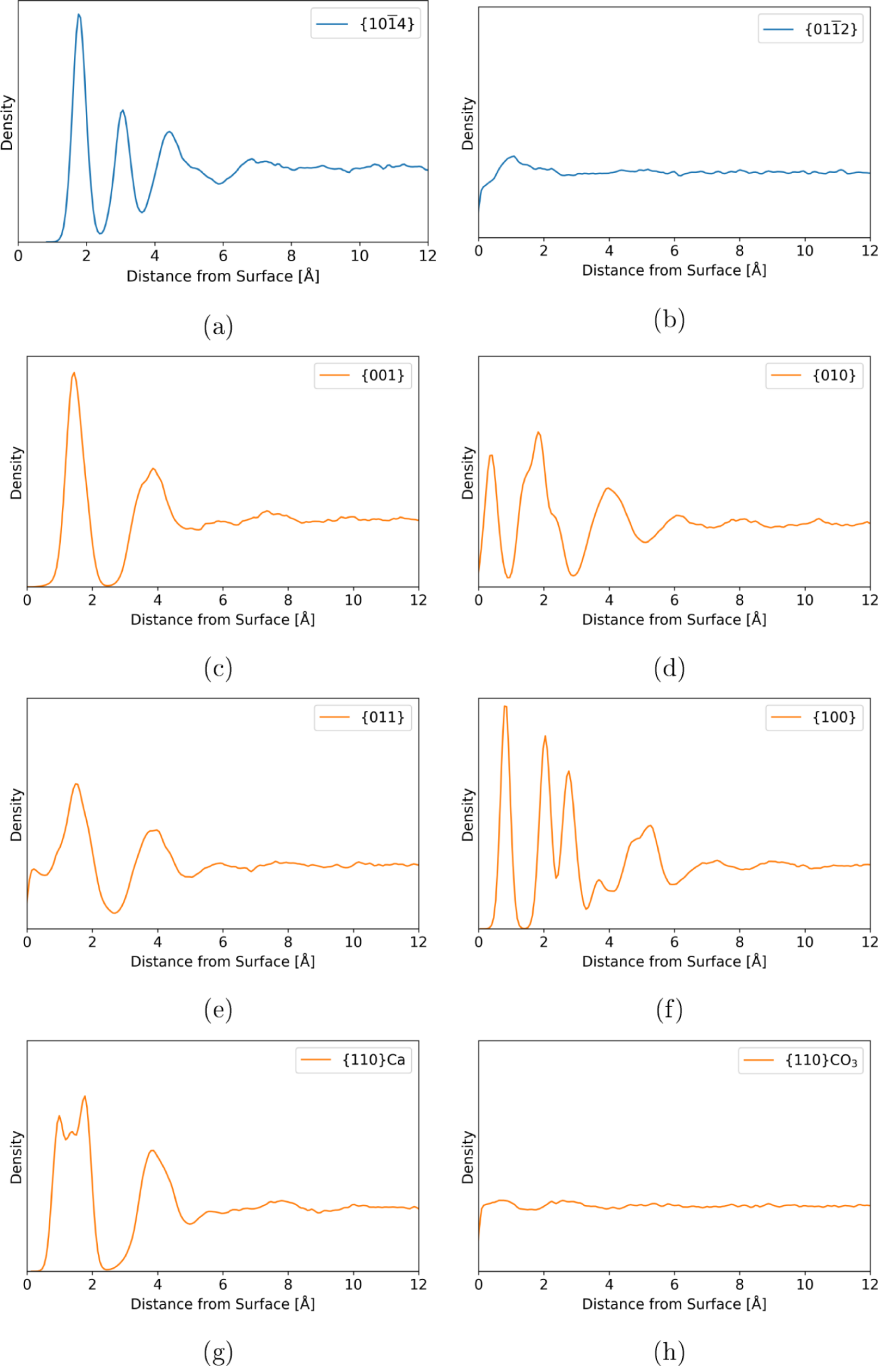
(a–h) *O*_W_ density profiles normal to the mineral surface. Both the calcite (blue) and aragonite (orange) interfaces are shown. Structured water layering is present at the interface of all but the two cases with the highest interfacial free energy, (b) and (h).

Figure 4 shows the 1D water density profiles normal to the surface plane out to 12 Å from the interface for all of the cases considered. The peak positions and relative sizes of the peaks for the {101̅4} interface are in good agreement with previous density profiles.^20^ In all cases, there is very little long-range order beyond 6 Å, and the water density is generally bulk-like. For the least stable cases, {011̅2} and {110}CO_3_, there is negligible ordering, even at the interface. These surfaces show larger surface relaxations that disrupt the regular crystal structure (Figure S2 (left)). This leads to a water density profile more akin to amorphous calcium carbonate surfaces.^64^ There is some semblance of structured water layers at the interface for all of the other surfaces. The width and height of the layers vary between the systems, suggesting different patterns of water density. There are also regions of zero (or near-zero) density present in some of the profiles, suggesting possible areas where water is entirely excluded. In contrast, on the aragonite {011} surfaces, we see a nonzero density at the interface. This is due to the almost stepped nature of the surface, which leaves carbonates pushing out into the solution, and therefore, some water molecules are brought closer to the surface (see Figure S2).

**Figure 5:**
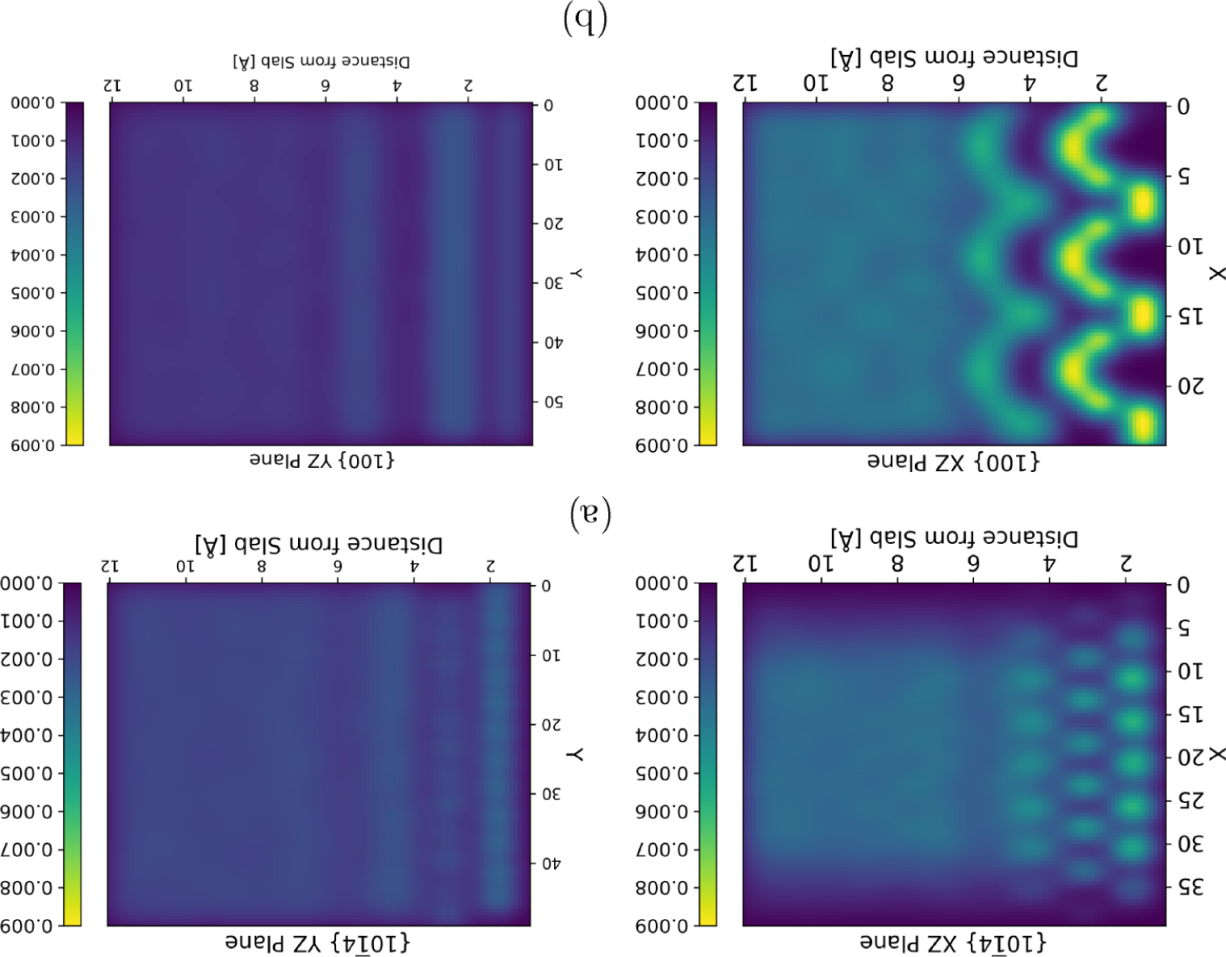
Density profiles for the calcite {101̅4} (a) and aragonite {100} (b) interfaces.

Some 2D density profiles for calcite {101̅4} and aragonite {100} are shown in Figure 5. The remaining surfaces along with visualizations of the crystal–water interface are recorded in Figure S2. The information from the 2D density profiles (*zx* and *zy* planes) supplements the conclusions from the 1D *z*-density profile. There is very strong ordering present in the {100} interface, particularly in the *xz* plane, which relates to its very large entropic penalty of formation. The space available around the ions at the surface of the {100} slab allows water to accumulate and order, as shown in Figure 5b. All of the aragonite interfaces (with the exception of the {110}CO_3_ interface) show a degree of ordering in both planes. For the calcite {101̅4} interface, there is a relatively strong degree of structuring shown in the *zx* plane, but there is comparatively little in the *zy* plane. This suggests that ordering within the planes is potentially important for entropy loss and that measuring the degree of order in only 1D does not provide enough information. Ordering in all dimensions should be considered.

Again, the two most unstable surfaces ({011̅2} and {110}CO_3_) show very little ordering in either plane. The atoms at the interface of these slabs have disorganized the most and show far less ordering in the liquid layers than other interfaces. These two surfaces have a large entropy loss associated with their surface energy, and we would speculate that this is due to ions partially dissolving into the solution leading to the formation of solvation shells in the water that will cause a significant entropy change but would not be observed with these visualization methods.

The ordering factor, *f*_order_ (eq 7), gives a simple but useful measure of how the water in the system is structured, values of which are presented in Figure S3. From the density plots in Figure 5, the {100} surface is the most ordered, which is reflected in the values obtained. Relative ordering in the other aragonite planes is harder to distinguish; the ordering factors lie closely together. The {011̅2} and {110}CO_3_ interfaces show the least structured water and thus the lowest values of *f*_order_. The low value of *f*_order_ for the calcite {101̅4} reflects the lesser ordering in the *zy* plane and the smaller difference in region density in comparison to those of the aragonite surfaces. Generally, the degree of ordering in the density plots correlates to the entropy contribution of the respective system.

**Figure 6:**
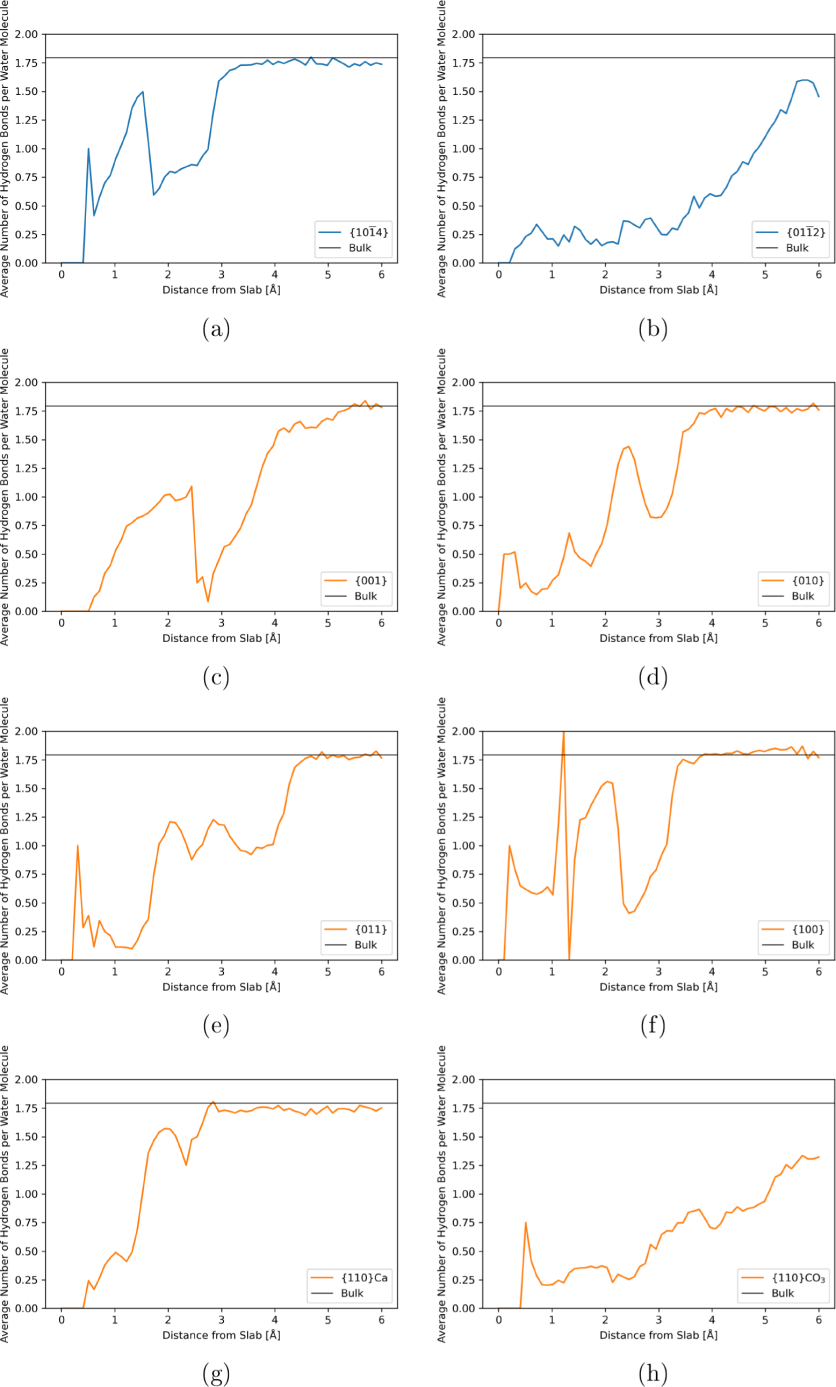
(a–h) Hydrogen bonding at the surface interface can reach up to 6 Å. The average numbers of hydrogen bonds per water molecule are calculated for 0.1 Å regions from the surface, and the position is determined by the donor atom’s coordinate. Each value is compared to that calculated for the bulk water system, which is given by the black line at a value of 1.795 hydrogen bonds per water molecule.

The variation of the average number of hydrogen bonds with the distance from the slab is shown in Figure 6. Close to the slab, the number is always lower due to the reduced availability of other water molecules to bond with. As the distance from the surface increases, the average number of hydrogen bonds roughly tends to that of the bulk, although noise from the data causes discrepancies in some cases. In general, the peaks and troughs in the graph correspond to the high and low water regions as seen in the density plots.

The {101̅4} surface shows relatively high degrees of hydrogen bonding due to the small distances (<5 Å between centers in the *x* direction) between high-density regions of water and between the layers of water perpendicular to the slab. It is likely that bonds can form in and between these regions. For the {001}, {010}, and {011} surfaces of aragonite, there are slightly larger gaps between water regions than exhibited in the calcite surface and much more distinct separation between the water layers. Therefore, the number of donor and acceptor oxygens available is lower, which is reflected in the lower average values. There is no definite pattern to be made between hydrogen bonds and the free energy of the surface or its enthalpic and entropic components.

The aragonite {100} interface is the only system where the average number rises above the bulk value; it then immediately drops to zero (this is partially a feature of the bin size of the analysis). This occurs in the densest regions at the surface, suggesting very strong ordering is happening there. This surface has the largest entropy loss, which would correlate with the strong ordering. Given the apparent ordering exhibited by the waters in the {110}Ca system, the average number of hydrogen bonds is higher than one might expect compared with the other aragonite surfaces. However, this particular system shows the two most distinct water layers at the interface with a structure that could favor hydrogen bonding across it, as the large peak occurs at the same position as the first water layer.

The most surprising results are those of the {011̅2} and {110}CO_3_ slabs. Since the water densities of the two systems are almost uniform and most “bulk-like”, one might expect that the hydrogen bonding would also be similar to that in the bulk. Both systems show very low amounts of hydrogen bonding, and the average value does not converge to that of the bulk until approximately 8 Å from the interface, whereas the other systems reach the bulk value between 3 and 5 Å. This again supports the possibility that solvation shells form around loose surface ions and significantly disrupt the hydrogen bonding network.

### 3.6 Temperature Effects

#### 3.6.1 Bulk Calcite and Aragonite

Free energies and enthalpies of the bulk phases of calcite and aragonite have been computed at a range of temperature values, as shown in Figure S4 and Table S1. Calcite is more stable at all temperatures; there is no crossover point at which aragonite becomes the more stable form. Although aragonite is less stable than calcite, there is very little difference (∼0.02%) between the free energies. The difference between the calcite and aragonite enthalpies is even smaller, and both increase at approximately the same rate. Experimentally, the magnitude of the aragonite enthalpy is greater than the calcite enthalpy.^63^ The force-field used in this work is, however, known to get the enthalpy stabilities the wrong way round.^20^ Our calculated difference between the two phases is of the same magnitude as the experimental results.

#### 3.6.2 Surface Energies

Experimentally, the nucleation of calcium carbonate at temperatures of 70 °C and above leads to the formation of aragonite before calcite. A possible explanation for this phenomenon would be if the aragonite nuclei were more stable than the calcite nuclei due to lower surface energies at these temperatures. In this scenario, the aragonite nucleus could form and grow more readily than the calcite nucleus. The more stable aragonite nuclei would then consume the solute ions, preventing calcite nuclei from reaching their critical size and therefore inhibiting the formation of the most thermodynamically stable phase.

Our results have highlighted that, at 300 K, aragonite interfacial free energies are higher than the calcite {101̅4} surfaces due to the greater entropy penalty associated with their formation, which we ascribed primarily to the loss of entropy due to the ordering of the water molecules in the surface vicinity. As we heat these systems, there are multiple effects that would be expected. First, the bulk water will become less ordered with fewer hydrogen bonds. This would suggest that there could be a greater entropy penalty for surface formation. Second, the water with more ambient heat may be less structured and ordered at the surface. This would suggest a smaller entropy penalty with surface formation. Third and finally, more disorder at the interface may lead to a larger enthalpy penalty with formation. Therefore, we can expect changes in the interfacial free energy of the surfaces that could alter the nuclei stability.

**Figure 7:**
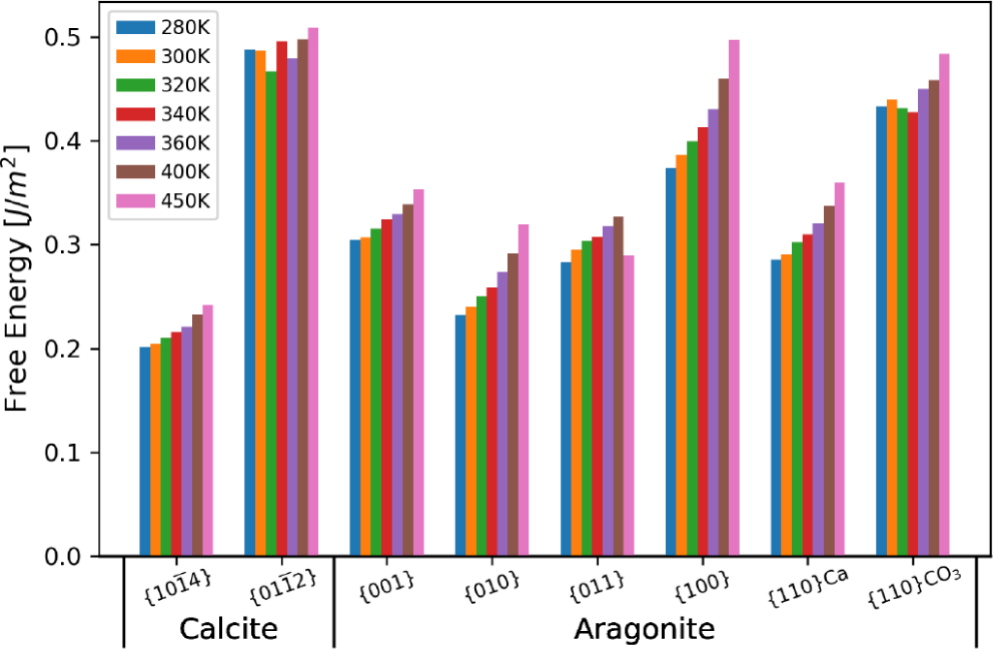
Interfacial free energies for all surfaces across various temperatures for calcite and aragonite surfaces.

As with the calculations at 300 K, the interfacial free energies of each surface at all temperatures were computed using TI. Enthalpies were then calculated from a single simulation, and the entropy of the system was determined as the difference in free energy and enthalpy, −*T*Δ*S* = Δ*F* – Δ*H*. The values are shown in Figure 7 with an enthalpy–entropy breakdown recorded in Table S2. In general, the free energies increase as the temperature increases. The relative energies and stabilities of the surfaces vary little across the temperature range. At all temperatures, the {101̅4} surface has the lowest free energy, and thus, calcite is the most stable polymorph. As with the bulk free energies, the gap between the lowest calcite surface, {101̅4}, and the lowest aragonite surface, {010}, increases with the temperature. The general trend of increasing interfacial free energy with temperature is followed by the {011̅2} and {110}CO_3_ surfaces, but these show a greater variation. We attribute this to a lack of water structuring at the surfaces, as the entropy is instead largely lost due to the disorder of the surfaces and partial solvation of ions. The {011} aragonite surface also shows a drop in the interfacial surface at 450 K, which is due to the onset of extreme disorder in the surface.

The fraction of free energy due to entropy is not constant across temperature changes (Table S2). In general, the entropy component increases, suggesting that the more disordered bulk water is dominating the entropy loss whereas the enthalpy generally decreases, which suggests that the interfaces are able to make more stable interactions with the solution. The entropy fraction of the free energy initially increases, reaching a maximum between 350 and 450 K, before decreasing at higher temperatures. The most stable aragonite surface, {010}, also has the highest entropy contribution with a maximum entropy fraction greater than 76% of the free energy at 380 K. Surfaces with lower entropy contributions, {101̅4} calcite and {001} aragonite, exhibit a maximum entropy contribution at a lower temperature than surfaces with lower entropy contributions and higher free energies, {011̅2} and {110}CO_3_.

#### 3.6.3 Crystal Morphologies

Using the interfacial free energy values calculated at different temperatures, the expected equilibrium crystal morphology is calculated by using the Wulff construction. For calcite, the {101̅4} surface is always the most stable with free energy values considerably lower than those of the {011̅2} surface. Therefore, the calcite morphology does not change with temperature. The average interfacial energy of the crystal, γ_Nano_, will increase with temperature, but the shape factor does not change.

There is a small change in morphology with the temperature for aragonite. The expressed surfaces remain the same with some slight changes in the relative sizes of the {001}, {010}, {011}, and {110} surfaces.

The free energy of converting a calcite nanoparticle to an aragonite nanoparticle as a function of the number of formula units, *n*, shows that for all temperature values considered, calcite is the dominant polymorph regardless of the number of formula units. We can therefore conclude that the formation of aragonite at higher solution temperatures is not due to a shift in interfacial energies favoring the aragonite nuclei.

#### 3.6.4 Water Ordering

The water densities change little in their distributions as the temperature varies. Examples are given in Figure S5 for the {101̅4} surface of calcite and the {010} surface of aragonite across a range of temperatures. There is some alteration in the distributions, and the patterns present become less defined with temperature, as would be expected with the greater movement of atoms. This is the case for all of the surfaces.

**Figure 8:**
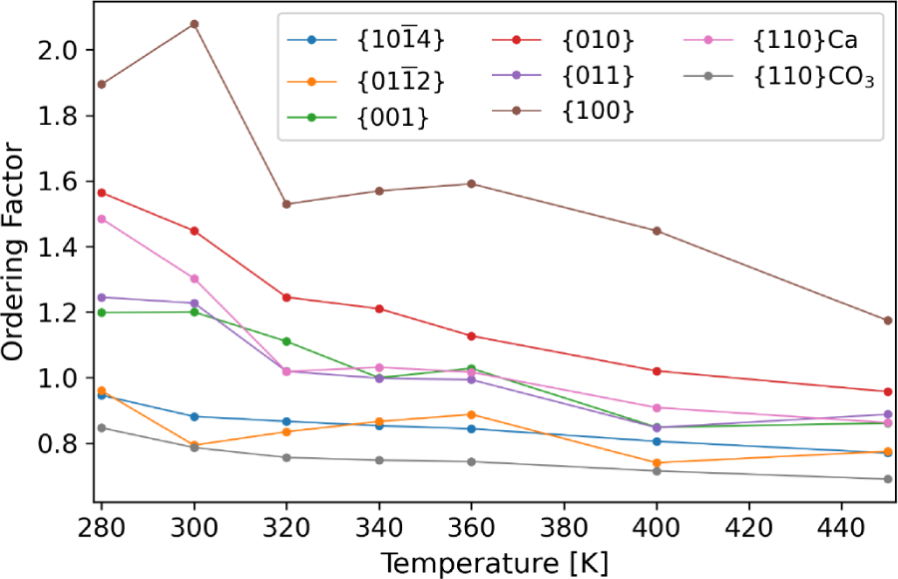
Variation of order factor for different surfaces of calcite and aragonite across the temperature range.

Although there is little visible change with temperature, the previous method of calculating order can again be implemented to quantify the structuring in the liquid and determine if it changes across our systems. Since the interfacial waters are compared with a slab of bulk water further out in the system, how bulk water varies with temperature is also considered. Details of how the ordering factor varies for each surface are shown in Figure 8. There is a general decrease in the ordering with temperature, as one might expect. The relative ordering between surfaces fluctuates a little between the surfaces as the temperature is increased, but in general, the major differences are maintained. For example, {100} remains the most ordered and {101̅4} remains less ordered than all the aragonite surfaces with the exception of {110}CO_3_.

It is important to note that although our ordering value reflects the atomic structuring of water seen in the density plots, we are considering only the positional density of the oxygen atoms. Dynamical features of water structuring are not included in our factor. Despite its difficulty, the development of a more thorough ordering factor would be an important follow-up to the work presented here.

## 4 Conclusions

We have presented a set of accurate interfacial free energies for calcium carbonate mineral surfaces using the force-field of Raiteri.^33^ Our results demonstrate that entropy makes a significant contribution to these interfacial energies. The size of the entropic contribution to the free energy varies considerably across the surfaces, and a uniform entropy correction is not sufficient for crystal/liquid interfacial energy calculations.

There is a clear distinction between the two polymorphs, with the metastable aragonite surfaces having, on average, a much higher entropic contribution than the calcite surfaces. This difference leads to the greater stability of the calcite polymorph at all crystal sizes compared to aragonite, explaining previous confusion when examining the enthalpy alone.

Distinct water ordering at the interface is clear from the 1D density profiles. Observing the density of water in all three dimensions highlights further ordering occurring in the planes of the system. Additionally, the surface characteristics influence the water structuring and thus the entropy lost in the system. Rough surfaces produce little ordering in the liquid. Surfaces with exposed cations and sufficient space available to trap water at the interface create a highly ordered structure. The hydrogen bonding in the system also demonstrates the differing restrictions imposed on the water molecules. There is a strong resemblance between the hydrogen bonding profile and the layered structure of water at the corresponding interface. It is well-known that there are structured water layers at the calcite {101̅4} interface, but our results show that more order is found at the aragonite interfaces. Our simple approach to quantifying the water ordering provides a tool to quantify the density profiles of the 3D liquid and provides a guide to the magnitude of the entropic loss in the formation of the surface.

Although the free energy of the surfaces increases with temperature, the relative stability of the surfaces remains broadly the same. Calcite, specifically the {101̅4} surface, is always the lowest in energy and, thus, the most stable. Therefore, the formation of aragonite at higher solution temperatures cannot be explained using interfacial energies and the stability of nuclei.

The entropy contribution, however, varies significantly with the temperature. The maximum entropy contribution for a surface is around 76%, and the lowest contribution is around 30%. For all surfaces, the maximum entropy contributions to the free energy occur between 370 and 410 K. When considering the discrepancy between the melting and boiling points of our chosen water model and the known values for water, it is possible that this temperature range coincides with the temperature range at which aragonite formation is primarily seen.

Given that the entropy makes up the majority of the free energies of aragonite surfaces, there is a large proportion that can be altered by disrupting the water structure in the system. If the entropies of the aragonite surfaces are reduced such that the total free energy is lower than that of calcite, then aragonite would become the more stable polymorph for small nanoparticles/nuclei. Since the maximum entropy proportions occur at the aragonite precipitation temperature range, this could be one reason for the temperature effects on calcite/aragonite stability. However, we have considered only pure water in our systems. In reality, there will be a substantial amount of ions in the system: individual Ca^2+^-CO_3_^2–^ and Na^+^-Cl^+^ ions from the initial solutions. These additional ions could have further effects on the interfacial free energies and stabilities of the surfaces not revealed here. There are also issues around the structure of any nanoparticle where relaxations could lead to surface curvature changes and different line energies than assuming a perfect Wulff morphology. Clearly, there is also potential competition from other phases, such as ACC and vaterite, for stability at small sizes. We know, however, that at some particular size, a clear crystalline structure will form, and the simulations suggest that calcite will dominate due to a combination of bulk and surface free energy terms. The enthalpy and entropy contributions do not show a simple trend across all surfaces. There is a clear distinction between the contributions to the calcite and aragonite surfaces. This could influence the polymorph selection and nucleation rates. Given the high sensitivity of these rates to interfacial free energies, it is imperative that the entropy is included in interfacial free energy calculations.

## Supporting Information Available

Figure S1: Bulk and surface water enthalpies; Figure S2: Density profiles for water; Figure S3: Ordering factor for water; Figure S4: Free energy for calcite and aragonite; Figure S5: Water density at different temperatures; Table S1: Free energy for calcite and aragonite; Table S2: Interfacial free energy for different temperatures (PDF)
