# Supplementary material for: Surface Free Energies and Entropy of Aqueous CaCO3 Interfaces
Source: Langmuir. 2025 Mar 18;41(12):8092–105. doi: 10.1021/acs.langmuir.4c04738 (PMC11966754; doi:10.1021/acs.langmuir.4c04738)
Supplement: Supplementary file 1 — la4c04738_si_001.pdf [file la4c04738_si_001.pdf]

Supplementary Information for Surface Free Energies and Entropy of Aqueous $CaCO_3$ Interfaces

Emma Armstrong,[†,‡] Stephen R. Yeandel,[†] John H. Harding,[†] and Colin L. Freeman[*,†]

†*Department of Materials Science and Engineering, Sir Robert Hadfield Building, University of Sheffield, Mappin Street, Sheffield, S1 3JD, UK*

‡*Information School, The Wave, University of Sheffield, 2 Whitham Road, Sheffield, S10 2AH, UK*

E-mail: C.L.Freeman@Sheffield.ac.uk

SI Figure 1

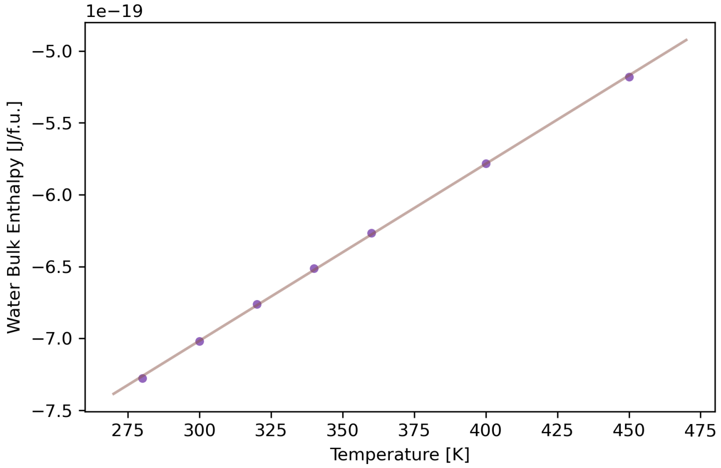

(a)

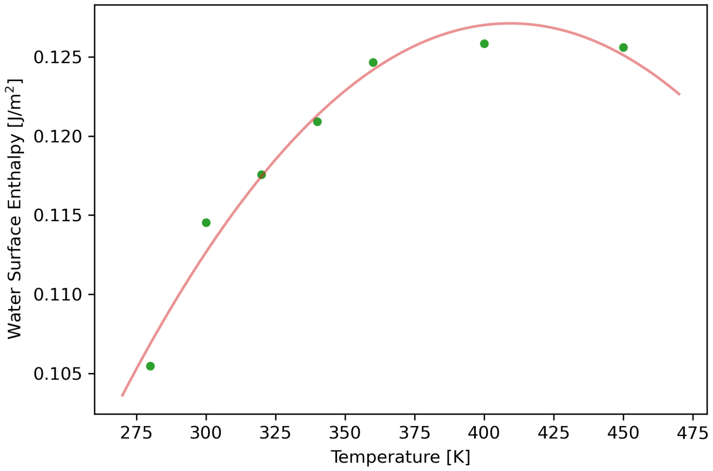

(b)

Figure 1: *Calculated bulk water enthalpies* (a) *and water surface enthalpies* (b) *from 280 to 450 K.*

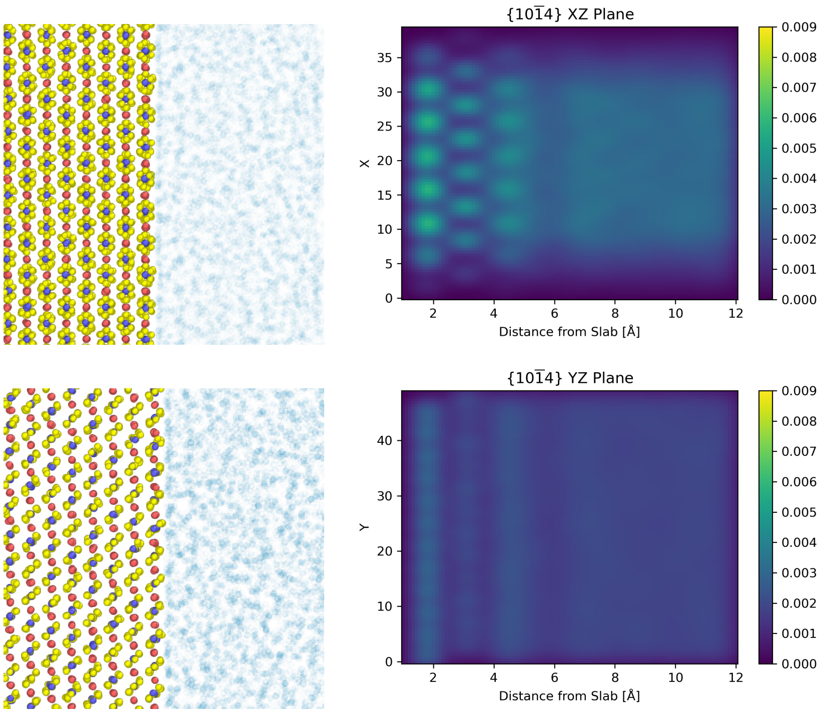
{10$\bar{1}$4} XZ Plane
X
Distance from Slab [Å]
{10$\bar{1}$4} YZ Plane
Y
Distance from Slab [Å]

(a)

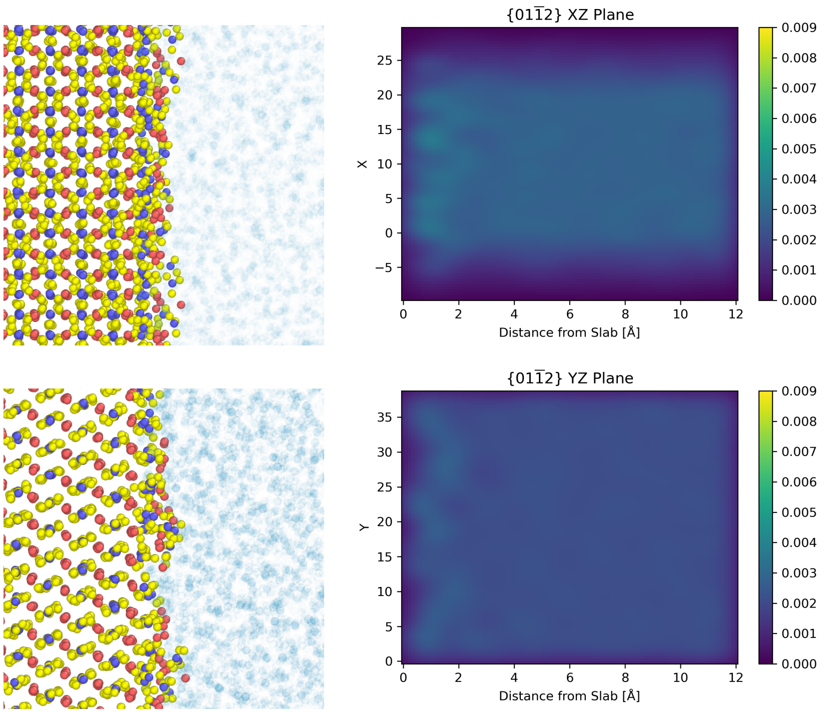
{01$\bar{1}$2} XZ Plane
X
Distance from Slab [Å]
{01$\bar{1}$2} YZ Plane
Y
Distance from Slab [Å]

(b)

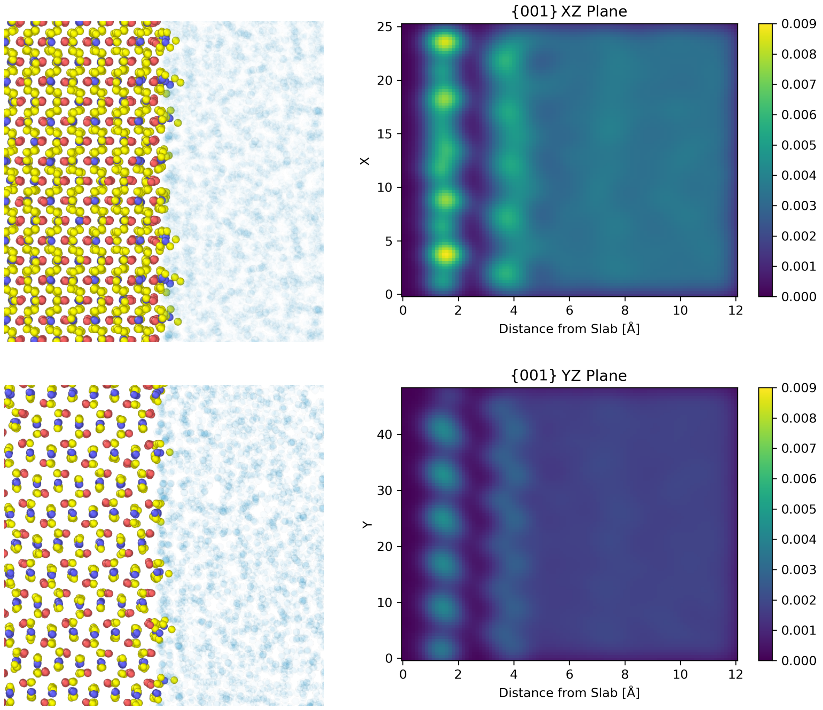
{001} XZ Plane
X
Distance from Slab [Å]
{001} YZ Plane
Y
Distance from Slab [Å]

(c)

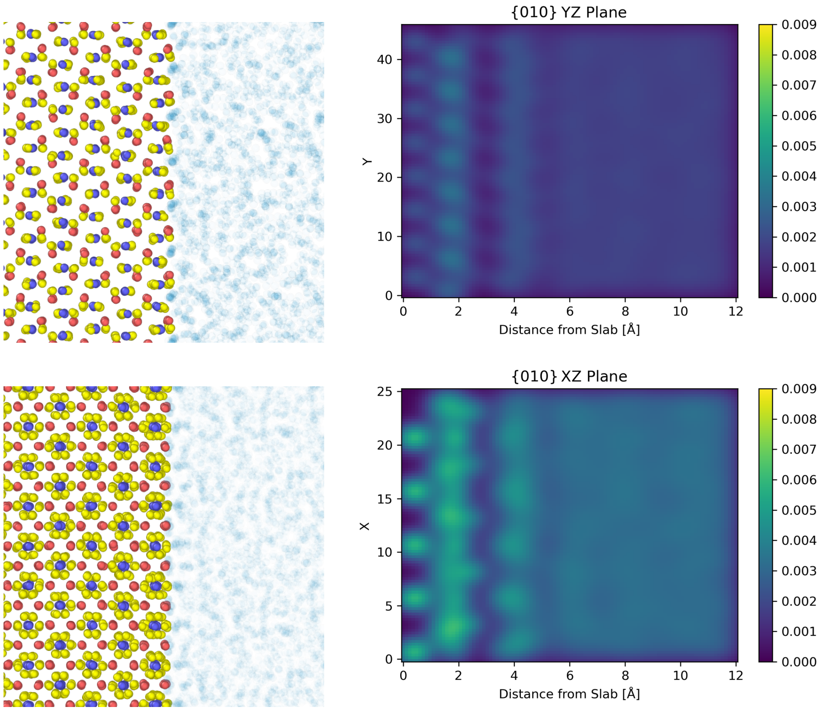
{010} YZ Plane
Y
Distance from Slab [Å]
{010} XZ Plane
X
Distance from Slab [Å]

(d)

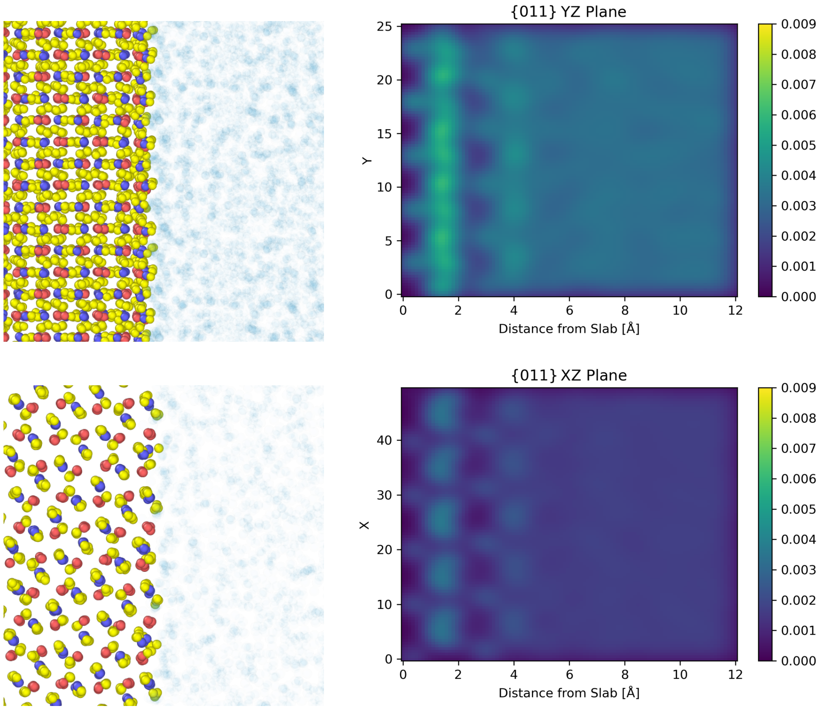
{011} YZ Plane
Y
Distance from Slab [Å]
{011} XZ Plane
X
Distance from Slab [Å]

(e)

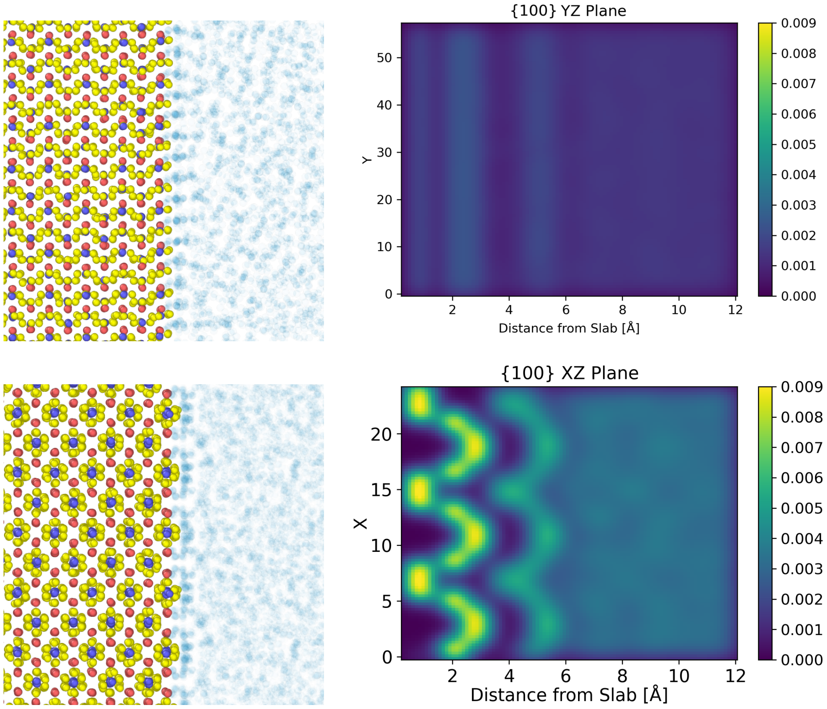
{100} YZ Plane
Y
Distance from Slab [Å]
{100} XZ Plane
X
Distance from Slab [Å]

(f)

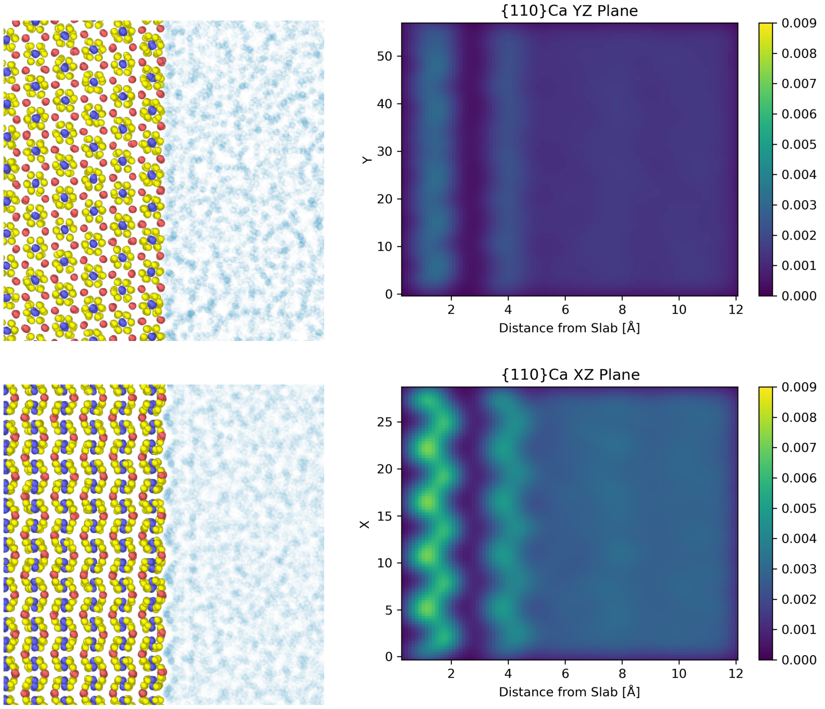

(g)

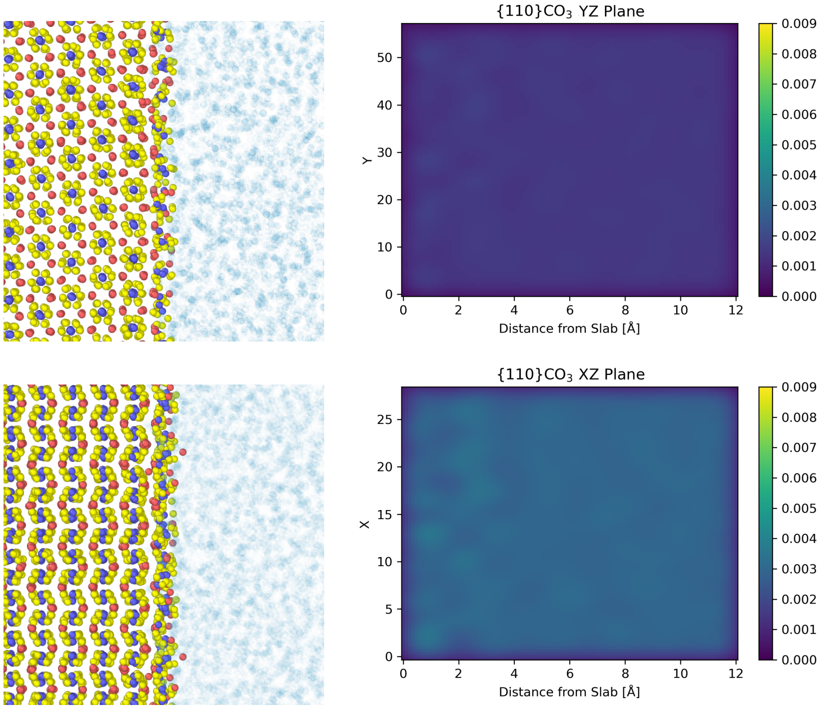

(h)

Figure 2: *Density profiles for the calcite* (a)-(b) *and aragonite* (c)-(h) *interfaces alongside visualisation of the crystal-water interface. Note that the scale differs between the interface visual and the density plots.*

SI Figure 3

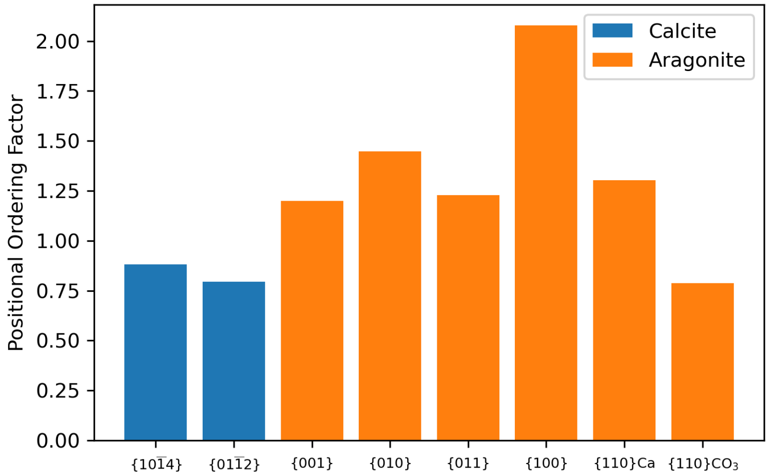

Figure 3: *Positional ordering factor for interfacial waters in both the calcite and aragonite systems. The higher the factor the greater the structuring of the waters at the interface. The calculated values generally describe the relative order expressed in density plots, encapsulating the ordering in all three dimensions.*

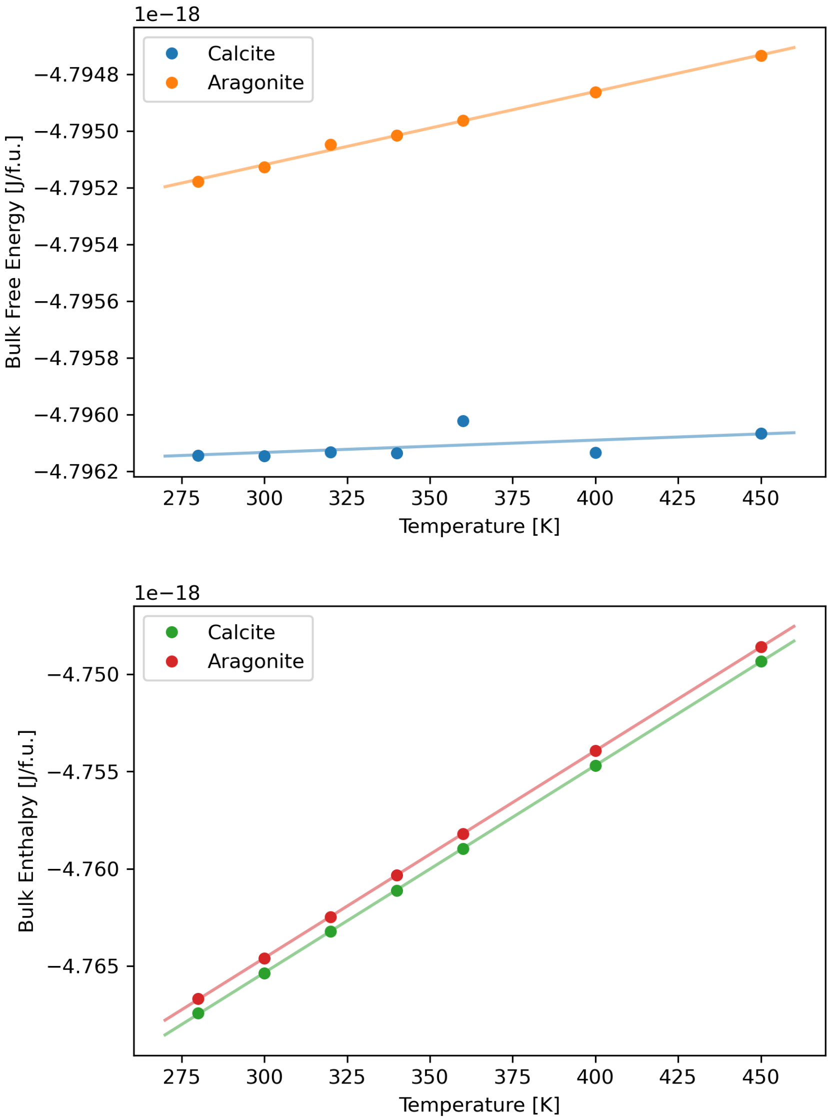

(a)

Figure 4: *Free energy* (a) *and enthalpy* (b) *cohesive energy values (with respect to the Einstein crystal) calculated for bulk calcite and aragonite via the Einstein crystal method for various temperatures.*

SI FIgure 5

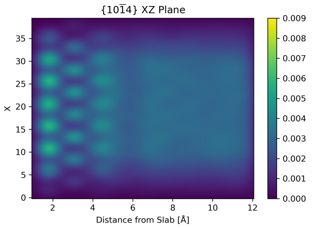

(a) 300 K

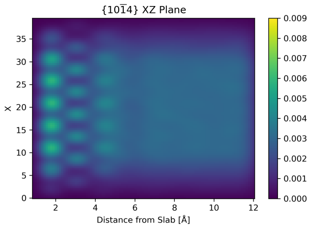

(b) 360 K

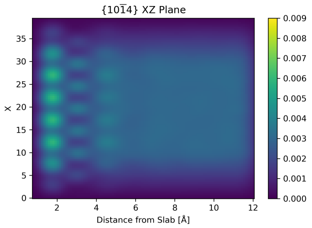

(c) 450 K

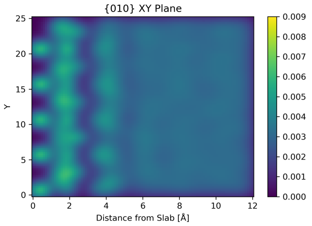

(d) 300 K

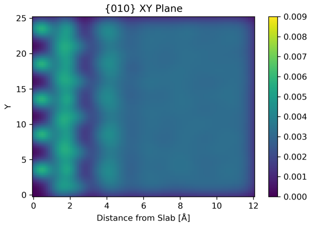

(e) 360 K

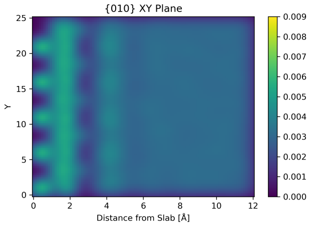

(f) 450 K

Figure 5: *Examples of water density plots for calcite* $\{10\bar{1}4\}$ *surface* (a) - (c) *and aragonite* $\{010\}$ *surface* (d) - (f) *at a sample of temperatures.*

SI Table 1

Table 1: *Values for the bulk free energy and enthalpy for calcite and aragonite with temperature increase.*

	Calcite		Aragonite	
T [K]	ΔF [J/F.U. $\times 10^{-18}$]	ΔH [J $\times 10^{-18}$]	ΔF [J/F.U. $\times 10^{-18}$]	ΔH [J $\times 10^{-18}$]
280	-4.79614	-4.76741	-4.79518	-4.76668
300	-4.79615	-4.76536	-4.79513	-4.7646
320	-4.79613	-4.76321	-4.79505	-4.76247
340	-4.79614	-4.76112	-4.79502	-4.76032
360	-4.79602	-4.75897	-4.79496	-4.7582
400	-4.79613	-4.7547	-4.79486	-4.75393
500	-4.79607	-4.74934	-4.79473	-4.74861

SI Table 2

Table 2: *Values for the interfacial enthalpy and entropy × temperature for calcite and aragonite with temperature increase.*

Calcite								
	T [K]	280	300	320	340	360	400	450
$\{10\bar{1}4\}$	ΔH [J m^{-2}]	0.142	0.133	0.130	0.126	0.130	0.132	0.163
$\{10\bar{1}4\}$	-TΔS [J m^{-2}]	0.060	0.071	0.080	0.090	0.091	0.100	0.079
$\{01\bar{1}2\}$	ΔH [J m^{-2}]	0.0.342	0.325	0.264	0.274	0.257	0.242	0.263
$\{01\bar{1}2\}$	-TΔS [J m^{-2}]	0.146	0.162	0.203	0.222	0.223	0.256	0.246
Aragonite								
	T [K]	280	300	320	340	360	400	450
{001}	ΔH [J m^{-2}]	0.204	0.189	0.190	0.184	0.184	0.190	0.223
{001}	-TΔS [J m^{-2}]	0.100	0.118	0.125	0.140	0.145	0.149	0.130
{010}	ΔH [J m^{-2}]	0.093	0.084	0.073	0.068	0.066	0.074	0.097
{010}	-TΔS [J m^{-2}]	0.139	0.156	0.177	0.191	0.208	0.218	0.223
{011}	ΔH [J m^{-2}]	0.158	0.166	0.280	0.287	0.285	0.295	0.162
{011}	-TΔS [J m^{-2}]	0.125	0.129	0.164	0.168	0.182	0.188	0.188
{100}	ΔH [J m^{-2}]	0.187	0.171	0.159	0.150	0.144	0.145	0.173
{100}	-TΔS [J m^{-2}]	0.187	0.216	0.241	0.263	0.286	0.315	0.325
{110}Ca	ΔH [J m^{-2}]	0.165	0.152	0.153	0.149	0.147	0.153	0.177
{110}Ca	-TΔS [J m^{-2}]	0.121	0.139	0.150	0.161	0.173	0.184	0.183
{110}CO_3	ΔH [J m^{-2}]	0.277	0.255	0.222	0.208	0.218	0.182	0.221
{110}CO_3	-TΔS [J m^{-2}]	0.157	0.185	0.210	0.220	0.232	0.277	0.263
